# A Language Index of Grammatical Gender Dimensions to Study the Impact of Grammatical Gender on the Way We Perceive Women and Men

**DOI:** 10.3389/fpsyg.2019.01604

**Published:** 2019-07-10

**Authors:** Pascal Mark Gygax, Daniel Elmiger, Sandrine Zufferey, Alan Garnham, Sabine Sczesny, Lisa von Stockhausen, Friederike Braun, Jane Oakhill

**Affiliations:** ^1^Department of Psychology, University of Fribourg, Fribourg, Switzerland; ^2^Department for German Language and Literature, University of Geneva, Geneva, Switzerland; ^3^Institute of French Language and Literature, University of Bern, Bern, Switzerland; ^4^School of Psychology, University of Sussex, Brighton, United Kingdom; ^5^Department of Psychology, University of Bern, Bern, Switzerland; ^6^Institute for Psychology, University of Duisburg-Essen, Duisburg, Germany; ^7^Allgemeine Sprachwissenschaft, University of Kiel, Kiel, Germany

**Keywords:** grammatical gender, gender representation, index, typology, language comparison

## Abstract

Psycholinguistic investigations of the way readers and speakers perceive gender have shown several biases associated with how gender is linguistically realized in language. Although such variations across languages offer interesting grounds for legitimate cross-linguistic comparisons, pertinent characteristics of grammatical systems – especially in terms of their gender asymmetries – have to be clearly identified. In this paper, we present a language index for researchers interested in the effect of grammatical gender on the mental representations of women and men. Our index is based on five main language groups (i.e., grammatical gender languages, languages with a combination of grammatical gender and natural gender, natural gender languages, genderless languages with few traces of grammatical gender and genderless languages) and three sets of specific features (morphology, masculine-male generics and asymmetries). Our index goes beyond existing ones in that it provides specific dimensions relevant to those interested in psychological and sociological impacts of language on the way we perceive women and men. We also offer a critical discussion of any endeavor to classify languages according to grammatical gender.

## Introduction

The way we perceive women and men in society is partly grounded in the way we speak or write about these two groups. As such, language acts not only as a vehicle for beliefs, but also as a tool that builds them. For example, ordinary people, as well as the media, communicate gender-stereotypical expectations with regard to gender-appropriate behaviors and roles for women and men, and such communication might lead individuals to define themselves and behave in accord with these expectations (e.g., Hannover, 2002; Sczesny et al., 2018). Consequently, one can easily argue that language biases gender representations through its communicative functions. However, language contributes to biased gender representations in other ways, with its intrinsic characteristics creeping into the way we perceive women and men.

There are different ways that this can happen. For example, at a syntactic level, word order may signal to readers or listeners specific semantic and societal hierarchies (e.g., Hegarty et al., 2016; Kesebir, 2017). Referring to *a woman and a man* or to *a man and a woman* is not perceived as being the same, and the resulting biased representations – toward the first person mentioned – have been well documented (Hegarty et al., 2016). Others have also documented biased uses of verbs and nouns when people refer to women or men. Typically, verbs denoting agency (i.e., more active) are more present in the immediate neighborhood of the word *men* [e.g., *men* (verb)] than the word *women*, and nouns and adjectives (i.e., more passive) more present in the immediate neighborhood of *women* (e.g., Formanowicz et al., 2017). These are some examples of the way language might constrain the way we think of women and men.

In this paper, however, we wish to concentrate on another characteristic feature of language that has kept psycholinguists particularly busy for the last two decades: grammatical gender. Most research on grammatical gender and gender representations has reflected the extent to which formal features of a language, such as the existence and number of grammatical gender categories (i.e., gender marking of pronouns, and/or nouns), may contribute to (biased) gender-related representations.

According to Dixon (1982), a language possesses grammatical gender when the following three criteria are met: (1) all nouns in a language are grouped into classes, (2) there is grammatical agreement between nouns and their dependent words or elements (e.g., articles, adjectives, verbs), and (3) the class membership of nouns shows a considerable semantic correlation with sex.^1^ This definition is more restricted than the one used by Corbett (1991) in his seminal book on gender, which did not include the third criterion. Dixon’s definition, which includes the sex dimension, appears to be more suitable for psycholinguistics research interests, because this research is often concerned with questions of gender-fairness, linguistic reference to and mental representations of women and men (for reviews see Stahlberg et al., 2007; Gabriel and Gygax, 2016; Sato et al., 2017; Gabriel et al., 2018). Sex-based grammatical gender systems are common in Indo-European languages, yet the reasons why these systems have emerged are not clear (Corbett, 1991; Foundalis, 2002).

Although grammatical gender systems vary between languages – and this paper presents an index of some important differences lacking from previous indexes – they also share some characteristics that have been shown to greatly affect readers’ and speakers’ mental representations of women and men. An example of a characteristic commonly shared across languages (English, French, German, etc.) is the multiple meanings of the masculine form, used when referring to animate beings. In these gendered (e.g., French, German) and semi-gendered languages (e.g., English, for which this feature only applies to pronouns nowadays), the masculine form tends to be used either *specifically* – referring exclusively to men – or in a so-called *generic* way – when there are female and male referents, or when the gender of referents is unknown or irrelevant. Interestingly, the dual meaning of masculine forms is often grounded in historical androcentric (and sexist) pressures (Gabriel et al., 2018). For example, in English, the singular and non-gendered *they*, used for several centuries in English literature, met with fierce criticism by 19th century androcentric prescriptive grammarians, who – following an earlier drive to impose the sex-indefinite *he* – saw the masculine form as the *worthier* one (Bodine, 1975). In French, in the 17th century, grammarians deemed it important to establish the masculine form as the dominant one, as they felt that men were simply nobler than women (Viennot, 2014). Until the 17th century, it was not uncommon to refer to a group composed of women and men by using pair-forms, meaning both female and male versions [e.g., *les auteurs et autrices*… (male and female authors)]. In German, masculine nouns have been promoted as having the ability to refer generically to both sexes only from the beginning of the 20th century (Doleschal, 2002: 59). Formerly, women and men – namely feminine and masculine forms referring to them – had been treated separately by grammatical description, and masculine forms were not described as having both a specific and a generic meaning (Doleschal, 2002). Thus, in all the languages with grammatical gender that we discuss in our database, there is a potential bias in favor of the masculine forms. Note that – although they would constitute interesting languages to compare to – we are not aware of any European language with a similar potential feminine bias.

Psycholinguistic investigations of how readers derive gender from the masculine form mostly show that its alleged *generic* meaning is rather difficult to activate (e.g., Gygax et al., 2012). For example, in a series of experiments in French, Gygax et al. (2012) showed that people have trouble considering a person described with a female kinship term (e.g., a sister) as belonging to a group of people when the group was referred to with the masculine form (e.g., *musiciens* n.m. “musicians”). This effect was also present when participants were explicitly asked to consider the masculine form as a generic one. In a recent cross-linguistic study in German and Italian, Horvath et al. (2016) also showed that the use of the masculine form only (as opposed to the use of both feminine and masculine words) generated representations that were more strongly male (i.e., a higher percentage of men in a profession).

Others have also looked at lexical access of gendered nouns, in comprehension and production. Among them, some have examined different asymmetries between the masculine and the feminine forms (e.g., Beatty-Martínez and Dussias, 2019), whilst others have examined the underlying routes (e.g., form-related or lexically based routes) to access grammatical gender in speech production (see Wang and Schiller, 2019 for a review). Still, whether explicitly or implicitly, most studies on individual grammatical gender languages suggest that their findings are generalizable to other languages with *similar* grammatical features. Several cross-linguistic comparison studies have demonstrated this generalizability (see Esaulova and von Stockhausen, 2015, or Gygax et al., 2008). Some studies have also suggested that languages bearing *different* grammatical gender features may display differences in the ways that speakers of these languages mentally represent the world in terms of gender (see Sato et al., 2013, for a comparison of French and English). Some authors have interpreted these differences as being illustrative of the impact of language on thought (e.g., Sato et al., 2016), in line with Slobin’s (2003) *Thinking for Speaking* hypothesis, for example.

However, most cross-linguistic comparisons of grammatical gender effects on mental representations have documented interesting variations. Yet, the grammatical gender systems under investigation are not always described in detail, at least in terms of similarities and differences, and existing indexes do not always provide the adequate dimensions to do so (especially when the focus resides in the way women and men are perceived). Cross-linguistic comparisons will remain useful for documenting the effect of language on thought (and on social constructs), but a more fine-grained analysis of the grammatical gender systems under investigation is required. Most studies on the topic have concentrated on existing taxonomies (e.g., Corbett, 1991; the Gender across Languages Project: Hellinger and Bußmann, 2001-2003; Hellinger and Motschenbacher, 2015; Fedden et al., 2018 for non-canonical gender systems), although some characteristics of grammatical gender systems, such as those presented in this paper, may be more relevant for future psycholinguistic investigations.

In the present paper, we present a non-exhaustive index of 15 grammatical gender systems (i.e., Chinese, Czech, Danish, Dutch, English, French, German, Italian, Norwegian, Polish, Rumanian, Russian, Slovak, Spanish, and Swiss German), based on work that has been already conducted (or could easily be conducted) and on dimensions that we identified as relevant for psycholinguistic research. In accordance with our goal to provide data for research on gender biases, our index focuses only on gender-related information and does not document other differences between these systems. We first present the dimensions chosen, along with their justifications, as well as a comprehensive table (see Supplementary Table S1) on the language samples. The list of languages chosen is obviously not exhaustive, and we do hope that additional languages will be categorized using our classification system.

## The Language Index of Grammatical Gender Dimensions

When establishing the data for the *Language Index of Grammatical Gender dimensions*, we followed an *a priori* grouping of several gender system types based on features that are known for a broad range of languages. We excluded universal features such as the existence of lexical gender words (e.g., *woman*, *father*, *male*, *female*) or the possibility of combining lexical gender elements with other nouns (as in English *girlfriend*, *male teacher*). Such forms appear to exist in most languages and therefore do not help to differentiate between languages. Importantly, our index not only complements existing taxonomies of grammatical gender (e.g., Dixon, 1982; Corbett, 1991) – and therefore helps to classify languages according to grammatical gender –, but also offers new insights into particular language biases (often toward favoring masculine forms) that may be of particular interest to those examining the psychological and sociological impacts of language on the way we perceive women and men. As such, and to the best of our knowledge, we offer a new taxonomic perspective on grammatical gender.

For the purpose of the present paper, we identified five different language groups, based on previous gender system descriptions (e.g., Corbett, 1991; Hellinger and Bußmann, 2001-2003; Hellinger and Motschenbacher, 2015). Even though languages in the first and second groups are very similar in many respects, we present them as two distinct groups, as only languages in the first group make a systematic distinction for human nouns between masculine and feminine forms. This distinction is highly relevant for research of the way gender distinction affects our representation of women and men.

1. Grammatical gender languages (e.g., French, Spanish, Czech, German) are languages in which personal (i.e., human) nouns (French *l’enseignant*, *l’enseignante* “the teacher”, *le fils*, *la fille* “the son,” “the daughter”) as well as inanimate nouns (Spanish *la mesa* n.f. “the table,” *el despacho* n.m. “the desk”) are classified for gender. These nouns control agreement of various other lexical categories such as determiners, adjectives or pronouns. Gender assignment is mostly semantically arbitrary for inanimate nouns, whereas the grammatical gender of human nouns shows considerable correspondence with the sex of the referent (or gender identity; see note #2). However, in some cases, the grammatical gender of nouns denoting human referents is different from their lexical gender (German *das Mädchen* n.n. “the girl,” Czech *to děvče* n.n. “this girl”). In such cases, one can observe agreement according to grammatical gender (especially when the satellite elements are close to the noun) as well as agreement according to the to the gender of the referent (when such elements are more distant) (ex. German *Das Mädchen n.n., das pron.n. ich kennengelernt habe, heisst Eva. Es pron.n./Sie pron.f. ist aus Deutschland.*, “The girl that I’ve met is called Eva. She is from Germany.”). In other cases, nouns denoting humans may be used to refer to women and men (French *la personne* n.f. “the person,” *l’individu* n.m. “the individual”). The number of such hybrid names varies across languages (see Corbett, 2015, for a detailed account of hybrid nouns). Such examples should be avoided in experiments testing grammatical gender because they represent exceptions with respect to the functioning of gender systems.
2. Languages with a combination of grammatical gender and natural gender (e.g., Norwegian, Dutch) have grammatical gender distinctions for inanimate nouns as well as for some personal nouns. In such cases, gender generally relates to the sex or gender identity of the referents. Contrary to languages such as German, Italian or French, where human nouns are often differentiated between masculine and feminine forms, the majority of human nouns are not formally distinguished between masculine and feminine forms. They can therefore be used for female and male referents without being linguistically differentiated. In this respect, these languages are closer to natural gender languages like English. For example, these languages have nouns equivalent to the English *teacher*, *doctor*, *neighbor*, etc., that are not formally marked for gender. Pronouns usually express the sex of the referent or the gender identity of a human referent.
3. Natural gender languages (e.g., English) don’t classify inanimate nouns according to different genders. Most personal nouns behave similarly, meaning that they are not specified for sex or gender identity (e.g., *teacher, child, politician*). Personal pronouns distinguish between female and male forms, which are used to refer to male or female referents, according to their referential sex or gender identity (e.g., *my teacher* – *she*, *your teacher* – *he*).
4. Genderless languages with a few traces of grammatical gender (e.g., Oriya, Basque) most personal nouns (in words equivalent to *teacher*, *child*, *politician* in English) as well as personal pronouns are used for male or female referents without using distinct linguistic forms. A few gendered forms appear in nouns with gender suffixes or gendered adjective or verbal forms.
5. Genderless languages (e.g., Turkish and Finnish) are languages where most human nouns as well as pronouns are generally unspecified for gender. If there are distinctions in personal pronouns, they refer to other features than femaleness and maleness (e.g., Finnish *hän* “she/he” = human, animate vs. *se* “it” = inanimate). The structure of these languages therefore does not enforce the use of gender-marked forms, even though this information can be conveyed by lexical means, such as the Turkish *erkek* “man or male” or *kız* “girl.” Gender-suffixes may occur on human nouns: for example, the suffix *-tar* or *-tär* may be added in Finnish to some words (mostly professions) to create feminine forms (e.g., *näyttelijä* “actor,” *näyttelijätär* “actress”). However, they are no longer used to create new forms.

In Section 1 of our table (see Supplementary Table S1) languages have been classified according to these five groups. When reading the table, it is important to bear in mind that in Section 1, only the fields that are relevant, depending on the category to which a language belongs, have been filled. Linguistic descriptions for the other subgroups of languages are marked as “not applicable.”

The next three sets of features – described in Sections 2 to 4 of the Table – are common to all languages and therefore always filled in (see Supplementary Table S1). They pertain to various aspects of linguistic structures, the lexicon and language use,^2^ and have not been described in detail in previous taxonomies, specifically:

Morphology (esp. derivation): What (classes of) words (esp. personal nouns and personal names) have formal features that can be attributed to (and may be interpreted in the light of) genders or gender identities? Which derivational processes are relevant and where may one find negative connotations attached to certain forms? In a language such as French, some feminine/female forms (names as well as nouns) are morphologically derived from masculine/male (e.g., *poète* n.m. > *poétesse* n.f., “poet”), alongside structurally symmetric pairs of feminine/female and masculine/male forms (e.g., *directeur* n.m., *directrice* n.f. “director”) or common gender forms like *extrémiste* n.f. and n.m., “extremist”). Some of the derived feminine forms may carry a negative connotation (such as the suffix -*ette* in *gendarmette* n.f. “female police officer.”

Masculine-male generics: Which masculine word forms are not used specifically to refer to male referents, but may be used with the intention to generically refer to (groups of) individuals whose referential/biological gender is irrelevant or unknown? In French, generically used forms are found both in nominal forms (e.g., *lecteurs* n.m. pl., “readers”) as well as in agreement targets such as determiners (*le* det.m., “the”), pronouns (*chacun* pron.m., “each”) or adjectives (*intéressé* adj.m., “interested”).

Asymmetries: What types of asymmetric forms or semantic features can be observed in the lexicon? For example, address terms may not be symmetrical between women and men (e.g., in English, the potentially sexist distinction between *Mrs* and *Miss* for women, while only one form, *Mr*., exists for men). Certain feminine/female (or masculine/male) counterpart forms for certain types of designations (e.g., occupational titles) may be absent from the lexicon (e.g., in French the lack of corresponding forms for *médecin* n.m., “medical doctor” or *sage-femme* n.f., “midwife.” Other asymmetries, for example, can be found in morphology, semantic connotations related to masculine feminine equivalent forms, or in various types of derogatory meanings attached to certain forms.

Note that in some cases more fine-grained distinctions based on usage have not been exhaustively documented in the table (see Supplementary Table S1) for practical reasons. For example, a given form may exist, but its usage may be infrequent or fading. We still qualify it as *present* but urge that researchers interested in these particular features should always carefully control for its usage. For each feature, the following classification has been used:

– Present: a given feature is obligatory or very common in the language.
– Partially present: there are examples of this feature in the language, but they are exceptions, rather than rules.
– Absent: the feature does not appear in the language. Note that this tag was only used in reference to usage in Sections 2–4. When cells in the table concern other groups of languages in Section 1, these are filled in with the indication *not applicable*.

## Discussion

Psycholinguistic investigations of the way people perceive gender have shown different biases associated with the particulars of grammatical gender. Not surprisingly, since many languages possess grammatical gender, these investigations have been conducted across a wide range of languages. However, between language comparisons – as rich as they may be – face intrinsic questions of legitimate comparability. In the present language index, we present different grammatical gender dimensions that might be of special interest for those interested in cross-languages comparisons in the way grammatical gender constrains our mental representations of women and men.

However, constructing a language index raises some important issues that also need to be taken into consideration in order to document how grammatical gender is encoded across languages. While the classification of languages into one of the five main categories that we established (genderless, natural gender, etc.) is globally straightforward, even though some intermediate cases may arise, many issues arise for the more specific questions that are raised for all languages in Sections 2 to 4 of our index. One such issue is the necessity to determine whether some features are truly productive in a language. This question can hardly be answered based on the intuitions of native-speaking informants alone, as it requires the use of quantitative analyses. This implies that for every feature in every language, a correct estimation of its prevalence would require extensive studies of language use in corpus data. Conducting such empirical analyses is beyond the scope of our index. While conscious of the limitations of our approach, we had to content ourselves with an estimation of usage provided by native speaker informants that we divided into three intuitive categories (i.e., no examples, only a few examples come to mind, many examples). These categories provide an estimation that should therefore be treated with caution, and are best used as a starting point for researchers who are interested in one particular aspect of gender differences.

Another limitation of our index is that the usage of feminine forms has evolved over the past decades in many languages. As a result, many forms that are attested may now be falling out of use. For example, the use of the word *le minister* to designate a female (government) minister in French is now declining, following an official decision from the French government in 1997 to feminize occupation names for women (Cerquiglini, 2018). Thus, even though some naming practices might be recognized by informants as existing in their language, it does not mean that they still correspond to current practices, or would not be recognized as sexist by its speakers. Here again, extensive studies of languages use that go well beyond the scope of our index would be needed to determine the nature of current practices.

Yet again, we believe that our index represents a useful starting point for researchers who want to investigate these questions. Another issue is that naming practices often vary from country to country, even when those countries share the same language. French is a case in point. While in France, the feminization of occupation names for women is a recent phenomenon, the use of feminine names was already current practice decades earlier in other French-speaking countries such as Canada, Belgium, and Switzerland.

Finally, our index contains a sample of 15 languages, representing mostly the Indo-European family. However, grammatical gender distinctions are widespread across the languages of the world. According to a recent typological sample, they occur in 40% of the world’s languages (Corbett, 2013a). From those, 75% have a gender distinction based on sex (Corbett, 2013b). Adding languages from other families that fall into this category would therefore bring valuable enrichments to our index, allowing us to move beyond Western cultural representations of sex and gender, as cultural differences have an impact of the representation of gender. For example, Corbett (2013b) reports that in Lak, a language spoken in the central Dagestan highlands, girls were not classified within the category of rational females, which for example applied to grown-up women, but in the category of other (non-male and non-female) animate beings. This classification led to an evolution of usage concerning the terms of address for young women. Using the gender marking for animate but not females when addressing young women became a sign of politeness. Aside from such anecdotal examples, documentation of gender-related usage for these languages is to a large extent lacking. We hope, however, to be able to enrich the present database in the future with more publications on languages for which gender-related usage can be collected.

## Author Contributions

PG prepared the first draft of the manuscript and coordinated the work among all authors. DE and SZ completed the index and collected the final data for it. AG worked on the final draft of the manuscript. SS and LvS initiated the project within the ITN Marie Curie framework. FB, JO, and AG created the first version of the index and organised the collection of the initial data for the languages presented in the Supplementary Material. All authors worked on their specific language to complete the index.

## Conflict of Interest Statement

The authors declare that the research was conducted in the absence of any commercial or financial relationships that could be construed as a potential conflict of interest.

## Footnotes

1 By the use of the term *sex* here, we refer to the normative category of *women* and *men*. In the following text, we will use the terms *sex* or *gender identity* for women and men referents.

2 Note that although we evaluate these features as pertaining or not to different languages in the table (see Supplementary Table S1, for a.xlsm version with macro for improving exploration of the table, see https://drive.switch.ch/index.php/s/zFSUSxtgIID56Ch), we focus on the French language merely to illustrate them here.
